# Early-life environmental exposures and childhood growth: A comparison of statistical methods

**DOI:** 10.1371/journal.pone.0209321

**Published:** 2018-12-17

**Authors:** Brianna C. Heggeseth, Alvaro Aleman

**Affiliations:** Department of Mathematics and Statistics, Williams College, Williamstown, MA, United States of America; University of West London, UNITED KINGDOM

## Abstract

There is a growing literature that suggests environmental exposure during key developmental periods could have harmful impacts on growth and development of humans. Understanding and estimating the relationship between early-life exposure and human growth is vital to studying the adverse health impacts of environmental exposure. We compare two statistical tools, mixed-effects models with interaction terms and growth mixture models, used to measure the association between exposure and change over time within the context of non-linear growth and non-monotonic relationships between exposure and growth. We illustrate their strengths and weaknesses through a real data example and simulation study. The data example, which focuses on the relationship between phthalates and the body mass index growth of children, indicates that the conclusions from the two models can differ. The simulation study provides a broader understanding of the robustness of these models in detecting the relationships between any exposure and growth that could be observed. Data-driven growth mixture models are more robust to non-monotonic growth and stochastic relationships but at the expense of interpretability. We offer concrete modeling strategies to estimate complex relationships with growth patterns.

## Introduction

There is a growing literature that suggests environmental exposure during key developmental periods in-utero could have long-term impacts on children. Early-life exposure to chemicals, such as phthalates and bisphenol A, found in household products may increase the risk of obesity development by disrupting hormonal processes that mediate childhood growth [1–3]. Estimating how and which factors modify adiposity growth is essential for possible public health interventions. Two commonly used methods for modeling longitudinal growth are linear mixed effects models and growth mixture models [4, 5]. Both models account for dependencies in repeated measures and are general enough to allow for non-linear growth, and baseline exposures are incorporated to explain growth with either a deterministic or stochastic mechanism. While motivated by childhood growth data from the Center for the Health Assessment of Mothers and Children of Salinas (CHAMACOS) study, we focus on the performance of these statistical tools and compare the performance of these two class of models to characterize the variability in BMI childhood growth patterns, their ability to estimate potential non-monotonic environmental exposure associations with non-linear growth, and their robustness to the nature of the exposure relationship mechanism [6].

Normal childhood body mass index (BMI) trajectories are J-shaped starting at age two, falling until the adiposity rebound which occurs around age 5 or 6 and then rising until age 18 [7]. Deviations from this healthy pattern have been observed for the children in the motivating data set based on the CHAMACOS birth cohort. One of the goals of the study is to determine whether in-utero chemical exposure is associated with the variability in growth patterns [8]. A variety of longitudinal methods have been used with this data set to address this goal, but they are often presented in isolation with no direct comparison of results obtained with different types of models [9–12]. We use this data set to highlight the potential differences in conclusions from mixed-effects and growth mixture models when trying to estimate the relationship between growth and any exposure.

One basic statistical method for measuring associations with growth is to estimate the relationship between exposure and the outcome at cross-sectional snapshots in time. However, this approach does not directly study the association with the change over time. Some of the earliest statistical methods to study change and development were developed by R. A. Fisher [13]. His ideas about random effects laid the foundation for the linear mixed effects model, a standard longitudinal model that accounts for longitudinal dependencies by incorporating subject-specific random effects in addition to assuming the covariance structure of the errors [4]. In this model, the relationship between the mean outcome over time and continuous baseline exposures are often encoded in practice with interaction terms between time variables and exposures. This results in the assumption of a linear relationship between exposure and the growth parameter and is used extensively in the literature on exposure and development [10, 11, 14, 15]. While the model can accommodate non-linear effects by adding interaction terms with squared exposure levels, it is not commonly used in practice. An alternative is to categorize a continuous exposure based on quantiles but modeling the full functional relationship is encouraged [16, 17]. This approach to modeling the effect modification on growth assumes a deterministic mechanism, in that a one-unit change in exposure has a fixed effect on the average growth rate.

The growth mixture model, which is less widely used, takes a fundamentally different approach to model the relationship between baseline covariates and growth [5, 18, 19]. We assume that the variability in the growth pattern can be modeled with a finite set of sub-groups, each with a unique mean growth pattern. The probability an individual follows a sub-group growth pattern is modeled based on the exposure level, so the effect modification is assumed stochastic in nature. While the linear mixed effects model is a special case of the growth mixture model when the number of groups is one, the two models encode the relationship between the shape of the growth curve and baseline exposures in two drastically different ways.

There is extensive literature about the statistical properties of both of these models, but there is not much discussion directly comparing the utility of the two methods when the nature of the mechanism and form of the effect modification is unknown. In this manuscript, we aim to fill this gap in the literature by providing a side-by-side comparison of linear mixed effects models with growth mixture models in order to provide statistical guidance to researchers studying the impact of baseline exposures on longitudinal outcomes such as childhood growth. We contrast the performance of these models in their ability to accurately estimate the relationship between growth curves and baseline covariates, understanding that both the growth and exposure relationship may be non-linear. In Section 2, we review the two models and present typical model specifications used in software. Then in Section 3, we analyze our motivating data set of in-utero phthalate exposure and subsequent childhood BMI to illustrate differences in inference and scientific conclusions on real environmental epidemiological data. In Section 4, we present a simulation study to compare the models in terms of capturing the relationship between baseline covariates and growth pattern. We end in Section 5 with conclusions about the methods and practical guidelines for the epidemiology researcher.

## 1 Statistical models

### 1.1 Linear mixed effects model

Let $y_{i}=\left(y_{i1},...,y_{in_{i}}\right)$ be a vector of repeated outcome measures on the *i*th individual observed at times $t_{i}=\left(t_{i1},...,t_{in_{i}}\right)$. In a linear mixed effect model, the expected outcome value can be written as *E*(**y**_*i*_|**u**_*i*_) = **X**_*i*_***β*** + **Z**_*i*_ **u**_*i*_, where **Z**_*i*_ is a subset of the *n*_*i*_ × *p* design matrix **X**_*i*_ for the *i*th individual, **u**_*i*_ is a vector of random effects, and ***β*** is a vector of fixed, unknown parameters. We assume Normality for the random effects, **u**_*i*_ ∼ *N*(**0**, **G**), as well as the outcomes such that **y**_*i*_|**X**_*i*_, **Z**_*i*_, **u**_*i*_ ∼ *N*(**X**_*i*_***β*** + **Z**_*i*_ **u**_*i*_, Σ_*i*_).

In order to explicitly define the relationship between baseline factors and the growth curve, we can parameterize the model using a two-level framework. For the first level, we let the outcome for the *i*th individual at their *j*th observation time equal $y_{ij}=x_{ij}^{T}\beta_{i}+\epsilon$ where $\epsilon_{i}=\left(\epsilon_{i1},...,\epsilon_{in_{i}}\right)∼N\left(0,\Sigma_{i}\right)$ and $x_{ij}^{T}$ includes time varying covariates including the basis for time. Then for the second level, the individual parameter vector can be expressed as a linear model of subject-specific baseline covariates, ***β***_*i*_ = ***γ*****w**_*i*_ + **u**_*i*_, where **w**_*i*_ is a design matrix with baseline covariates that modify the effect of **x**_*ij*_ and **u**_*i*_ ∼ *N*(**0**, **G**). We combine the two levels to get an aggregate model, where **X**_*i*_ includes main effects and interaction terms between baseline factors **w**_*i*_ and time variables in **x**_*ij*_ and **Z**_*i*_ is a design matrix that includes the variables that have random effects. We assume that a change in the value of a baseline factor in **w**_*i*_ produces a change in the growth curve through the parameter vector ***β***_*i*_. For this paper, estimation of this model through restricted or standard maximum likelihood was implemented with the *lme4* package in R [20].

### 1.2 Growth mixture model

In a finite mixture model, we assume there exists a finite number of sub-groups, each weighted by a group probability [5, 21, 22]. Assuming each group has a probability density function, the mixture density is defined as a weighted sum of *K* sub-group densities, $f\left(y_{i}|X_{i},Z_{i},w_{i}\right)=\sum_{k=1}^{K}\pi_{k}\left(w_{i}\right)f_{k}\left(y_{i}|X_{i},Z_{i}\right)$ where **Z**_*i*_ is a subset of the *n*_*i*_ × *p* design matrix **X**_*i*_ for the *i*th individual that have random effects, and the weights, $\pi_{k}\left(w_{i}\right)=e^{w_{i}^{T}\gamma_{k}}/\left(\sum_{l=1}^{K}e^{w_{i}^{T}\gamma_{l}}\right)$ are the probabilities of being a member of the *k*th group based on a vector of baseline covariates **w**_*i*_ where ***γ***_*K*_ = 0 [23]. With a continuous longitudinal outcome, the subgroup densities are often assumed multivariate Gaussian or a distribution that is robust to outliers such as a t-distribution [24]. For a growth mixture model, the group mean is based on a linear mixed effects model with conditional mean, ***μ***_*k*_ = **X**_*i*_***β***_*k*_ + **Z**_*i*_**u**_*i*_ where **u**_*i*_ ∼ *N*(0, **G**) [5]. Thus, the linear mixed effects model is a special case of the growth mixture model when *K* = 1.

Growth mixture models are often estimated with a maximum likelihood method via an iterative algorithm. The expectation-maximization (EM) algorithm treats the group membership as missing data and iteratively estimates group membership and group parameters. The EM algorithm is guaranteed to find a local solution under mild continuity conditions, but a global maximum may be attained through the use of a variety of initializations [25]. In this paper, estimation of the growth mixture model was completed with the *lcmm* package in R [26]. To choose the number of groups appropriate to explain the heterogeneity in the data, we fit the model with *K* = 2, ‥, 5 and select the model with the lowest Bayesian Information Criterion (BIC) as it has been shown to work well for mixture models in practice [27, 28].

### 1.3 Mean model

With any growth model, the model for the mean needs to be flexible enough to capture the average trajectory over time. The most popular functional form assumes linear growth, which may be adequate for data collected over a short period. A low-order polynomial such as a quadratic or cubic function allows some curvature. To accommodate more local deviations, a spline, which is a piece-wise polynomial function, provides more flexibility in capturing non-linear growth. In the context of a linear model, a spline can be represented as a linear combination of basis splines or B-splines [29, 30]. The flexibility of the mean is constrained by the degree of the polynomial and the number and location of the knots, or change-points. Knots can be chosen based on percentiles of observation time or using expert knowledge of the underlying process, but the complexity of the model comes at a cost of degrees of freedom [31]. The spline basis functions for a given set of knots and degree were calculated using the splines package in R [32].

## 2 Childhood growth data

### 2.1 Data

To illustrate differences between the two modeling approaches, we first analyzed the relationship between the phthalate concentrations in a mother’s urine during pregnancy and the subsequent body mass index development of the child from age 2 to 13 years old. The data come from a longitudinal birth cohort study, the Center for the Health Assessment of Mothers and Children of Salinas (CHAMACOS) study, which enrolled pregnant women from prenatal clinics serving the farmworker population in the Salinas Valley, California in 1999-2000. Of 601 women enrolled in the study, a total of 527 were followed through the birth of a singleton, live-born infant. In-utero exposures from urine and blood samples were measured as the mean levels measured at the end of the 1st and the 2nd trimester of pregnancy and anthropometric measurements were measured on the children about every 1-2 years. BMI was calculated as weight (*kg*)/height^2^(*m*^2^). The Institutional Review Board (IRB) of the University of California, Berkeley, approved all study activities. Informed consent was obtained from all mothers; oral assent was obtained from children beginning at age 7 and written assent at age 12. Further details of the study have been previously described [33].

For illustrative purposes, the exposure we focus on is monoethyl phthalate (MEP) biomarker, a metabolite of diethyl phthalate used in fragrances and personal care products. Phthalate measurements in urine collected during pregnancy were available for 436 mothers, and of those mothers, 333 children had at least three measures of BMI between 2 and 13 years of age. There have been previous reports of associations of prenatal MEP concentrations with increased BMI in childhood [12].

### 2.2 Methods

**Fig 1:**
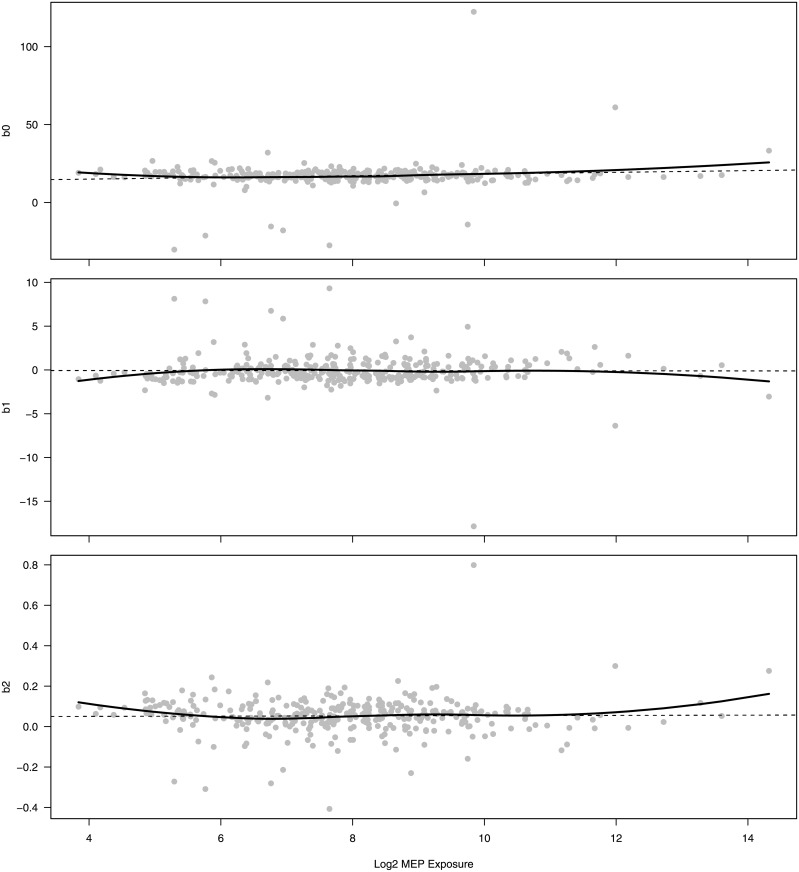
MEP exposure and growth parameters. MEP exposure during pregnancy (x-axis) and estimated growth parameters from individual quadratic model fits (y-axes) for the CHAMACOS data. Dashed line is a least squares line and the thick line is a smooth loess curve.

Prior to modeling, we estimated a quadratic BMI growth curve by age for each individual. Then, we used the derived parameter estimates to explore possible relationships between exposure and the level and growth of BMI over time. Fig 1 includes scatterplots of the maternal MEP exposure and the estimated coefficients from a quadratic function. The plots indicate there may be a non-monotonic relationship between maternal in-utero exposure and growth parameters, but the relationship is not clear.

Since BMI z-scores were not developed for repeated measures analysis, we analyzed BMI and stratified based on sex to allow sex-specific growth as well as exposure associations [34]. For the mean model, we chose a quadratic B-spline basis with one knot at age 9.5 years so as to minimize the sum of squared error modeling individual trajectories. The internal knot allows the BMI to have a slanted J-shape with an adiposity rebound and then linear growth in later childhood. In a linear mixed effects model, we allowed the MEP exposure to impact level and growth by including all pair-wise interactions with the spline basis variables. To account for dependence in repeated measures, we included a random intercept and random spline coefficients. In a growth mixture model, we included the MEP exposure in the group probability model to predict group membership probabilities and included a random intercept within the sub-groups. We used the BIC to choose the number of groups within the growth mixture model. To focus on the strength and weaknesses of the model, we only present unadjusted analysis without accounting for confounding variables, which would be necessarily if we were aiming to estimate a causal relationship. All analyses were completed using the splines, lcmm, and lme4 packages in R.

### 2.3 Results

**Fig 2:**
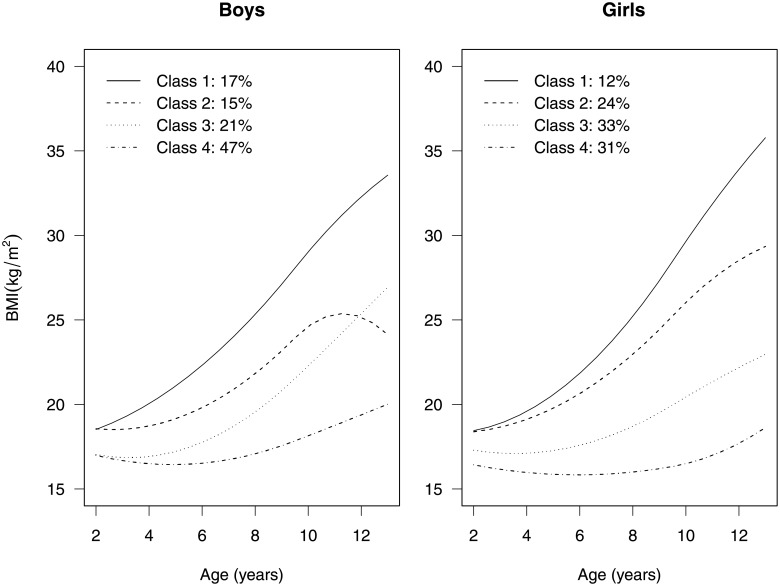
Group-based mean growth trajectories. Estimated group means for boys and girls from four-group growth mixture model fit to the CHAMACOS data.

**Fig 3:**
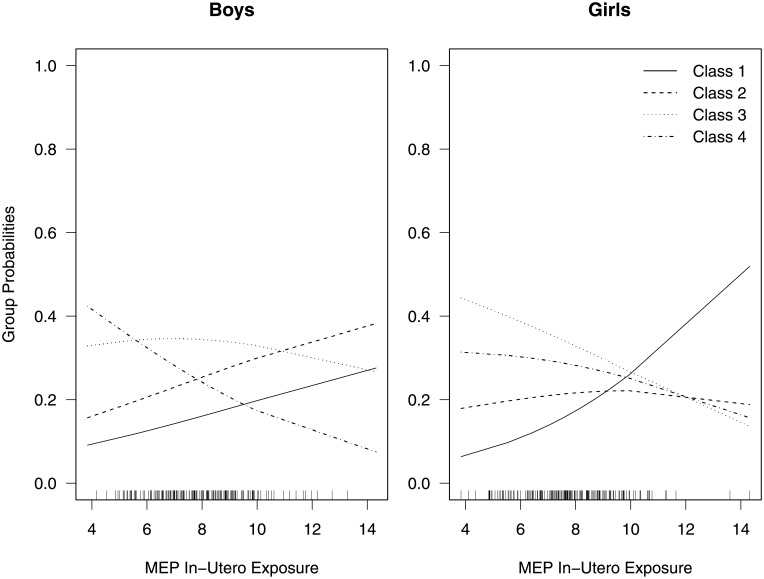
Group probabilities. Estimated group probabilities for maternal MEP exposure for boys and girls from a four-group growth mixture model fit to the CHAMACOS data.

For the mixture model, four groups were chosen to minimize the BIC. Fig 2 shows the four group means for boys and girls estimated from the growth mixture model. We note the variation in growth patterns within this group of children, ranging from a shallow U shape to a linear growth pattern between ages 2 and 13. Based on the estimated parameters of the group probability model (Table 1), MEP exposure has a slightly stronger stochastic impact on the growth pattern of boys than girls. We visualize the association between MEP exposure and the group probabilities in Fig 3. For doubling in MEP exposure, the ratio of the probability of being in class 1 and 2 vs. 4 increases by an estimated factor of 1.31 (90% confidence interval: 1.01, 1.70) and 1.28 (90% confidence interval: 1.01, 1.63), respectively, for boys. The point estimates for girls indicate an estimated multiplicative increase of 1.30 (90% confidence interval, 1.04, 1.65) and 1.07 (90% confidence interval, 0.86, 1.34) for a doubling of MEP exposure. Additionally, we do not see any evidence of differences in MEP exposure between class 3 and 4 for both boys and girls.

**Table 1:** Estimated ratios comparing class probabilities to the probability of Class 4 for a doubling of MEP and associated 90% confidence interval from estimated four-group growth mixture model fit to the CHAMACOS data, boys and girls separately.

Subgroups	Boy: Ratio (CI)	Girl: Ratio (CI)
Class 1: Log_2_ MEP Exposure	1.31 (1.01, 1.70)	1.30 (1.04, 1.65)
Class 2: Log_2_ MEP Exposure	1.28 (1.01, 1.63)	1.07 (0.86, 1.34)
Class 3: Log_2_ MEP Exposure	1.15 (0.92, 1.46)	0.95 (0.78, 1.18)
Class 4: Log_2_ MEP Exposure	1	1

CI, Confidence Interval

**Fig 4:**
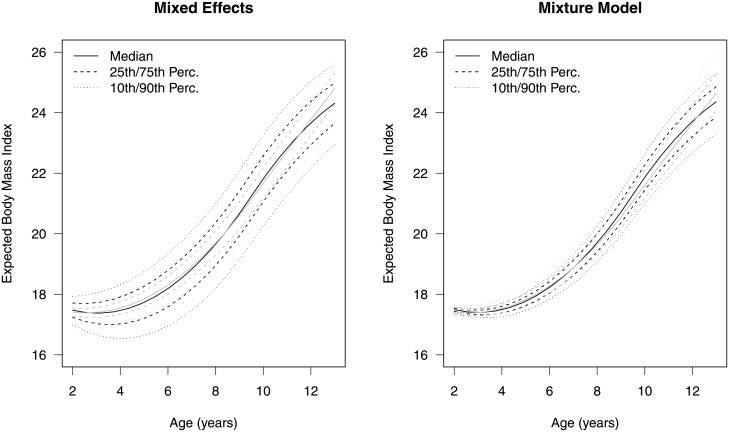
Model comparison. Estimated mean BMI for the 10th, 25th, 50th, 75th, and 90th percentiles of MEP for boys (dark) and girls (light), separately, based on a linear mixed effects model and a four-group growth mixture model fit to the CHAMACOS data.

The linear mixed effect model estimates suggests that doubling MEP exposure has a significant linear impact on the growth parameters for boys and girls through the interaction terms (Table 2). The effect modification is represented by the three interaction terms between the B-spline basis variables (*X*_1_-*X*_3_) and Log_2_ MEP exposure. For boys, two of the three are significantly different from zero and one of the three are significant for girls. Since the non-linear growth is not encapsulated in one parameter, we visualize the interactions by calculating the estimated mean BMI for a range of percentiles of MEP exposure for boys and girls for the linear mixed effects model (Fig 4). Similar to the growth mixture model, MEP exposure has a larger estimated effect on the growth pattern for boys in comparison to girls.

**Table 2:** Estimated fixed effect coefficients comparing class probabilities to the probability of Class 4 for a doubling of MEP and associated 95% confidence interval from the linear mixed effect model fit to the CHAMACOS data, boys and girls separately.

Variable	Boy: Coef (CI)	Girl: Coef (CI)
Intercept	15.85 (14.1, 17.6)	16.75 (15.0, 18.5)
*X*_1_ (B-spline basis for age)	-3.55 (-5.0, -2.0)	-1.93 (-3.4, -0.5)
*X*_2_ (B-spline basis for age)	2.03 (-1.6, 5.7)	2.77 (-0.9, 6.5)
*X*_3_ (B-spline basis for age)	4.89 (0.9, 8.8)	4.98 (1.0, 8.9)
Log_2_ MEP Exposure	0.22 (-0.01, 0.4)	0.08 (-0.1, 0.3)
*X*_1_*Log_2_ MEP Exposure	0.35 (0.2, 0.5)	0.19 (0.01, 0.4)
*X*_2_*Log_2_ MEP Exposure	0.55 (0.1, 1.0)	0.42 (-0.04, 0.9)
*X*_3_*Log_2_ MEP Exposure	0.31 (-0.2, 0.8)	0.40 (-0.1, 0.9)

Coef, Coefficient; CI, Confidence Interval

*X*_1_-*X*_3_ variables are the B-spline basis variables for age that flexibly model the mean BMI over ages 2–14. Interactions with MEP exposure allow linear effect modification of the growth trajectory.

The marginal means are similar between the two methods as evidenced by Fig 4, but the models differ in interpretations and resulting conclusions. The growth mixture model suggests the MEP has a significant association with the probability of growth pattern groups for boys and less so for girls and the relationship may be non-monotonic. The standard linear mixed effects model does not allow for stochastic or non-monotonic relationships and thus, we conclude there is some significant effect modification for both sexes and MEP exposure explains some variation in BMI growth. Interestingly, the linear mixed effect model suggests a larger effect of exposure on the mean when comparing the marginal expected values, but the growth mixture model highlights that the mean is not the only aspect of interest, the variability in growth is also important to note.

## 3 Simulation study

We designed a simulation study to compare the performance of the two models in estimating stochastic and deterministic effect modification. We generated data to mimic observed childhood BMI growth patterns and paired them with a variety of hypothesized environmental exposure associations. Then, we fit a series of linear mixed effect and growth mixture models to each data set to study how robust the models were in capturing the relationship with exposure if the model is misspecified based on mean model, exposure model, or exposure mechanism.

### 3.1 Data generation

**Fig 5:**
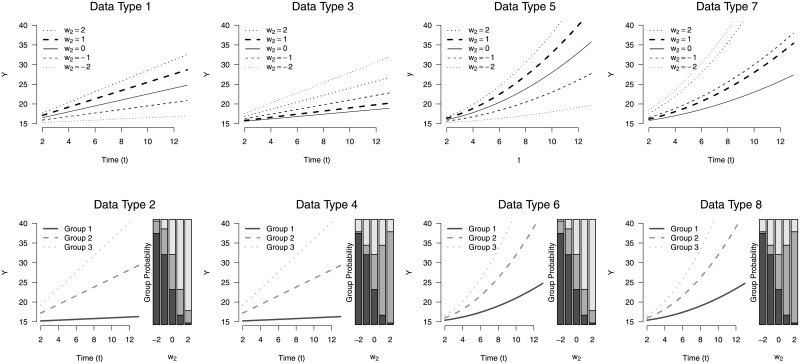
Conditional mean outcomes for simulation study data generation. For data types 1, 3, 5, and 7, the means are conditional on the quantitative exposure value of *w*_2_ and for data types 2, 4, 6, and 8, the means are conditional on the subgroups and the group probabilities for given exposure values are on the right.

We generated data based on both a linear BMI growth as well as a quadratic BMI growth curve similar to those observed in real data. We then created four data generating conditions based on hypothesized exposure relationships, differing in terms of functional form (monotonic or non-monotonic) as well as mechanism (stochastic or deterministic) of the effect modification. Fig 5 provides a graphical view of the conditional means for the eight data generating conditions for the simulated body mass index trajectories. Under each data condition, we generated 1000 training data sets of size *n* = 250, around the typical size of an environmental epidemiological study. We let *t*_*i*_ = (1, 3.25, 5.5, 7.75, 10) for *i* = 1, …, *n* be the observation times and generated a binary variable, *w*_1*i*_ = *Bernoulli*(0.5), to mimic a variable such as sex and a quantitative exposure of interest, *w*_2*i*_ = *N*(0, 1). For simplicity, this simulation does not model all potential confounders. For model comparison, we generated 1000 validation sets of the same size to test the out-of-sample predictive performance.

### 3.2 Model specifications

On each data set, we fit a series of linear mixed effects and growth mixture models under a variety of assumptions about the form of the mean growth and the relationships with exposure. We used two functional bases for the mean growth: linear and quadratic. We included only a random intercept or let all growth coefficients vary according to a multivariate Normal distribution with an unstructured covariance.

In the linear mixed effects models, we modeled the exposure association by allowing interaction terms between baseline exposure variables, *w*_1_ and *w*_2_, and the age variables. In the mixture models, we included the baseline exposure variables in the multinomial logistic model to model the group probabilities. This flexibly accommodates any non-monotonic dose-response relationships since heterogeneity in growth patterns is summarized by a discrete latent variable. For sake of comparison, we also fit models with a tertiary version of the exposure, *w*_2_, based on quantiles.

In total, we fit 16 models, which included every combination of model type, form of the mean, random effects, and form of exposure. The linear mixed effects models were fit using the *lme4* package in R and the growth mixture models were fit using the *lcmm* package in R, using the defaults for any assumptions not specified here and utilizing BIC as the criterion for choosing the number of groups, *K* [20, 26]. R code for the simulation study is publically available [35].

### 3.3 Model comparison

Selecting an appropriate model from a collection of candidate models is a key step of the scientific process. There are a variety of statistical tools to help us compare and select models. Information criterion such as BIC balance goodness of fit with model complexity [27]. Another tool to compare is the out-of-sample predictive performance of a model based on a validation set or cross-validation if data is limited [36]. Predictive performance is used here to check whether we are accurately capturing the relationships in the training data. In this study, we calculated the BIC from the trained models and the mean square predicted error on the validation data and averaged both quantities over the 1000 simulated data sets within each condition and model.

To compare the model’s ability to accurately estimate the relationship between the quantitative exposure and growth from these two different models, we calculated the estimated mean function and its first derivative on a uniform grid of values for time and the continuous baseline exposure, *w*_2_, keeping the other baseline variable fixed. We compared the estimates on the grid to the true expected value *μ*(*t*, *w*_1_, *w*_2_) = *E*(*Y*|*t*, *w*_1_, *w*_2_) and growth velocity $v\left(t,w_{1},w_{2}\right)=\frac{d}{dt}E\left(Y|t,w_{1},w_{2}\right)$ based on the data generating distribution.

We calculated the absolute error on the grid as $\delta \left(t,w_{1},w_{2}\right)=\left|\hat{\mu }\left(t,w_{1},w_{2}\right)-\mu \left(t,w_{1},w_{2}\right)\right|$ and the absolute velocity error on the grid as $\delta^{\prime }\left(t,w_{1},w_{2}\right)=\left|\hat{v}\left(t,w_{1},w_{2}\right)-v\left(t,w_{1},w_{2}\right)\right|$. To summarize the discrepancies in estimated values and velocity across time and baseline values, we estimated the mean absolute error (MAE) and mean absolute velocity error (MAVE) over the uniform grid, $\text{MAE}=\frac{1}{C}\int \int \delta \left(t,w_{1}=1,w_{2}\right)dtdw_{2}$ and $\text{MAVE}=\frac{1}{C}\int \int \delta^{\prime }\left(t,w_{1}=1,w_{2}\right)dtdw_{2}$ where C is the area of the rectangular domain region. In contrast to the mean squared prediction error, this measure does not account for the distribution of observation time and exposure, but rather focuses on the performance across a given domain. We limit our grid to a subset of the simulated domain, *t* ∈ [1, 9], *w*_2_ ∈ [−2, 2], and *w*_1_ = 1. The MAE and MAVE are then averaged over the 1000 simulated data sets within each condition and model.

### 3.4 Results

**Fig 6:**
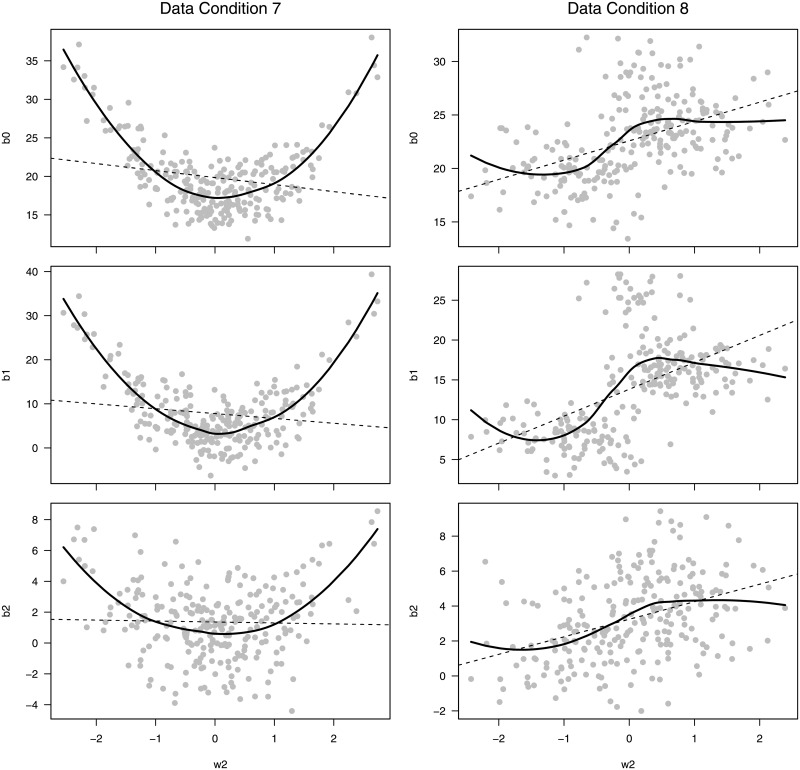
Simulated quantitative exposure, *w*_2_ (x-axis) and growth parameters from individual quadratic model fits (y-axes) for one data set under data condition 7 (quadratic growth with a deterministic, quadratic relationship between growth parameters and exposure) and data condition 8 (quadratic growth with a stochastic, non-monotonic relationship between growth pattern probabilities and exposure). Dashed line is a least squares line and the thick line is a smooth loess curve.

Derived variable analysis is useful for visually exploring the relationships in the data and to inform the model choice. Fig 6 provides a graphical analysis for simulated datasets under the last two data conditions labeled 7 and 8, which include non-linear growth and non-monotonic effect modification for exposure and growth, under a deterministic and stochastic generation process, respectively. For illustration, we derived estimated growth parameters for each individual trajectories using a quadratic function. Notice the clear quadratic relationship between *w*_2_ and the growth parameter under data condition 7. Under data condition 8 (stochastic mechanism), the non-linear relationship is not as obvious. There is a slight “S” shape in the scatterplot since the probability of growth parameters changes with exposure rather than the parameters themselves.

We present the results from the best linear mixed effects and growth mixture models with the continuous version of *w*_2_. We denote the mixed effects models with linear mean with random intercept as ME1, quadratic mean with random intercept as ME2, linear mean with random intercept and slope as ME3, and quadratic mean with random intercept and slope as ME4. Mixture models with the same mean and random effect specifications are denoted as M1-M4. Results from models with tertiary version of the exposure are available in supplementary information (Tables in S1, S2 and S3 Tables).

Table 3 provides the average BIC for each of the eight data conditions. The linear mixed effects model provides the best fit when the relationship between exposure and growth is deterministic and linear as the model matches the data generating distribution. However, if the effect modification is stochastic and/or non-linear, the growth mixture model provides a better fit evidenced by the lower BIC values.

**Table 3:** The BIC averaged over 1000 simulated data sets for a set of eight models under 8 different data conditions specified by the nature of the relationship, form of the growth patterns, and the form of the effect modification from the exposure. Smallest average BIC for each data condition is bold.

Nature	Growth	Exposure	ME1	ME2	ME3	ME4	M1	M2	M3	M4
D	L	L	6540	6540	6091	6111	6131	6150	**6076**	6086
S	L	L	7042	7041	6245	6264	**5985**	5997	6010	6009
D	L	NL	6725	6724	6152	6171	6192	6213	**6100**	6111
S	L	NL	7146	7145	6274	6294	**5983**	5999	5996	6007
D	NL	L	7772	7740	6674	**6402**	6872	6639	6672	6453
S	NL	L	7539	7286	7090	6455	6937	**6239**	6955	6244
D	NL	NL	7520	7491	6505	6352	6596	6492	6424	**6282**
S	NL	NL	7134	6894	6825	6346	6529	**5995**	6544	6016

ME, Mixed Effect Model; M, Growth Mixture Model; D, Deterministic; S, Stochastic; L, Linear; NL, Non-Linear

Models 1, 3 assume linear mean; Models 2, 4 assume quadratic mean.

Models 1, 2 use random intercept; Models 3, 4 use random slopes.

Table 4 provides the mean squared error (MSE) based on out-of-sample prediction on a validation set. A comparison of the average MSE between models for each type of data suggests that a growth mixture model can predict as well as or better than a linear mixed effects model. We see the greatest benefit when growth is non-linear and the exposure effect modification is non-linear. If there is a non-linear effect modification, stochastic or deterministic in nature, a growth mixture model can provide more accurate predictions over a linear mixed effects model with pair-wise interaction terms between the exposure and age variables.

**Table 4:** The MSE from a validation set averaged over 1000 simulated data sets for a set of eight models under 8 different data conditions specified by the nature of the relationship, form of the growth patterns, and the form of the effect modification from the exposure. Smallest average MSE for each data condition is bold.

Nature	Growth	Exposure	ME1	ME2	ME3	ME4	M1	M2	M3	M4
D	L	L	**15.3**	**15.3**	**15.3**	**15.3**	15.4	15.4	16	16
S	L	L	25.4	25.4	25.4	25.4	**25.1**	**25.1**	**25.1**	**25.1**
D	L	NL	18.6	18.6	18.6	18.6	17.6	17.7	**17.5**	17.7
S	L	NL	27.9	27.9	27.9	27.9	**27.4**	**27.4**	**27.4**	**27.4**
D	NL	L	35.5	**35**	35.5	**35**	35.7	35.3	37.9	35.8
S	NL	L	29.4	26.4	29.4	26.4	29.1	**26.1**	29.1	**26.1**
D	NL	NL	32	31.6	32	31.6	27.2	28	**23.7**	27.8
S	NL	NL	21.3	19.3	21.3	19.3	19.2	**17**	19.2	17.1

MSE, Mean Squared Error; ME, Mixed Effect Model; M, Growth Mixture Model; D, Deterministic; S, Stochastic; L, Linear; NL, Non-Linear

Models 1, 3 assume linear mean; Models 2, 4 assume quadratic mean.

Models 1, 2 use random intercept; Models 3, 4 use random slopes.

To supplement the information gained from BIC and MSE, Table 5 provides the mean absolute velocity errors on a grid so as to measure the accuracy of the effect modification estimation. Not surprisingly, the results continue to suggest the importance of accurately modeling the mean growth. Additionally, if the priority is estimating exposure’s association with growth rates, growth mixture models are more robust to misspecification in terms of nature and form of the exposure relationship.

**Table 5:** The mean absolute velocity error (MAVE) averaged over 1000 simulated data sets for a set of eight models under 8 different data conditions specified by the nature of the relationship, form of the growth patterns, and the form of the effect modification from the exposure. Smallest average MAVE for each data condition is bold.

Nature	Growth	Exposure	ME1	ME2	ME3	ME4	M1	M2	M3	M4
D	L	L	**0.75**	**0.75**	**0.75**	**0.75**	0.76	0.76	0.8	0.8
S	L	L	0.57	0.57	0.57	0.57	**0.55**	**0.55**	0.56	**0.55**
D	L	NL	1.64	1.64	1.64	1.64	**1.61**	1.62	1.62	1.63
S	L	NL	0.41	0.41	0.41	0.41	**0.33**	**0.33**	**0.33**	**0.33**
D	NL	L	1.09	**0.73**	1.09	**0.73**	1.1	**0.73**	1.16	0.78
S	NL	L	1	**0.74**	1	**0.74**	0.98	**0.74**	0.98	**0.74**
D	NL	NL	1.38	**1.08**	1.38	**1.08**	1.41	1.1	1.46	1.15
S	NL	NL	0.86	0.71	0.86	0.71	0.8	**0.62**	0.8	0.63

ME, Mixed Effect Model; M, Growth Mixture Model; D, Deterministic; S, Stochastic; L, Linear; NL, Non-Linear

Models 1, 3 assume linear mean; Models 2, 4 assume quadratic mean.

Models 1, 2 use random intercept; Models 3, 4 use random slopes.

A categorical exposure variable can be useful in detecting non-linear effect modification, but it may not provide an accurate estimate of the relationship. In fact, using the categorical exposure variable in a mixture model can hurt the performance measured by the four metrics presented (Tables in S1, S2 and S3 Tables).

This simulation study provides evidence to suggest that growth mixture models can accurately estimate the exposure relationship with growth even when the data were generated based on a deterministic setting with random effects. Additionally, a growth mixture model can more flexibly handle non-linear relationships between baseline exposures and growth. The same cannot be said for linear mixed effects models.

## 4 Discussion

Humans have informally studied change over time for generations, but useful methods for analyzing longitudinal data are much younger [37]. Two of the standard methodological approaches for longitudinal data, mixed effects models and marginal models estimated using generalized estimating equations, were introduced in 1980’s [4, 38]. There have been much discussion over the strengths and weakness of these two general types of models [39, 40]. On the other hand, there have been many innovative ways to combine mixed effect models with the more established class of models, mixture models [5, 41]. In particular, growth mixture models, which are a mixture of mixed effects models, have become more estimable through software and primarily used to find data-driven groups. The growth mixture model is a mixed effect model with only one group, if you disregard group membership model. If a group membership model is not used, then the two models can be considered nested. However, as we have shown, the group membership model is useful to approximate non-linear relationships between growth and baseline variables such as environmental exposures. To the authors knowledge, this is the first study comparing these two methods with a group membership model focusing on the relationship between baseline exposure and growth over time.

There is growing evidence of non-monotonic dose-response relationships for environmental exposures like those of endocrine disrupting chemicals but there are no standard guidelines for researchers to use when considering these complex relationships with growth. Our simulation study suggests that a growth mixture model with a stochastic exposure relationship performs as well or better than the deterministic mixed effects model with pair-wise interaction terms in accurately estimating the relationship. However, the improved performance with a growth mixture model comes at a cost of more complex interpretation as it models growth pattern probabilities rather than growth parameters.

While a categorical version of the exposure can be useful in detecting non-monotonic relationships, there is little guidance for choosing the best break points to optimize estimation. Based on our experience and the evidence provided, we suggest the following guidelines when estimating the relationship between a baseline exposure and growth over time.

As a first step, we suggest exploring the functional shape of the individual trajectories over time by graphing the trajectories of a subset of the individuals. Notice the variability in growth patterns in addition to the starting levels. Use nonparametric smoothing to visually contrast the estimated mean with a least squares line over time. This important step helps determine the appropriate functional basis over time and the variability in growth.

The next step is to explore exposure relationships by coloring individual trajectories based on percentiles of the exposure or using derived analysis. While not appropriate for making inferences, analysis of derived variables such as coefficients of polynomial projections can be a valuable tool to inform the selection of the class of models. Scatterplots for each coefficient highlights the different relationships that exist with the level and growth parameters.

If the growth pattern is a lower-order polynomial and there is a clear polynomial relationship between the exposure and parameters, then estimate a linear mixed effects model incorporating the observed relationships. If the time variable is centered at a meaningful time point, estimated coefficients of interaction terms can provide clear interpretations with respect to exposures impact on level, velocity, and acceleration at that time point.

If the growth pattern is not a lower-order polynomial or there is no clear polynomial relationship with the exposure, then we suggest utilizing a growth mixture model with an appropriate functional basis (e.g. polynomial or B-spline) and exposures in the multinomial logistic model to predict group membership probabilities. The complex model allows a more flexible relationship between exposures and growth. The possible association with exposure is summarized in ratios comparing group membership probabilities with the reference group. Consider the correlation between the level and growth within groups. If level and growth are not strongly correlated, then the level can be removed by subtracting the mean to focus on the exposure relationship with growth.

While the linear mixed effects model is a subclass of the more general growth mixture model when *K* = 1, using modeling selection criterion to choose between *K* = 1 and *K* > 1 does not adequately compare of these models as the incorporation of exposure data differs whether we have 1 or more than 1 group. We suggest fitting both types of models and using model selection criterion such as BIC and MSE to compare the goodness of fit and predictive power of the two types of models.

## Supporting information

S1 Table The BIC averaged over 1000 simulated data sets for a set of eight models using the tertiary version of *w*_2_ exposure variable under 8 different data conditions specified by the nature of the relationship, form of the growth patterns, and the form of the effect modification from the exposure. Smallest average BIC for each data condition is bold.
(PDF)

S2 Table The MSE from a validation set averaged over 1000 simulated data sets for a set of eight models using the tertiary version of *w*_2_ exposure variable under 8 different data conditions specified by the nature of the relationship, form of the growth patterns, and the form of the effect modification from the exposure. Smallest average MSE for each data condition is bold.
(PDF)

S3 Table The mean absolute velocity error (MAVE) averaged over 1000 simulated data sets for a set of eight models using the tertiary version of *w*_2_ exposure variable under 8 different data conditions specified by the nature of the relationship, form of the growth patterns, and the form of the effect modification from the exposure. Smallest average MAVE for each data condition is bold.
(PDF)
