# Supplementary material for: Early-life environmental exposures and childhood growth: A comparison of statistical methods
Source: PLoS One. 2018 Dec 17;13(12):e0209321. doi: 10.1371/journal.pone.0209321 (PMC6296561; doi:10.1371/journal.pone.0209321)
Supplement: S2 Table — Smallest average MSE for each data condition is bold. (PDF) [file pone.0209321.s002.pdf]

Supporting Information

To complement the results in the manuscript, we present the simulation results for the eight models using the tertiary version of w_2 rather than as a continuous variable. We denote the mixed effects models with linear mean with random intercept as ME1, quadratic mean with random intercept as ME2, linear mean with random intercept and slope as ME3, and quadratic mean with random intercept and slope as ME4. Mixture models with the same mean and random effect specifications are denoted as M1-M4.

S2 Table. The MSE from a validation set averaged over 1000 simulated data sets for a set of eight models using the tertiary version of w_2 exposure variable under 8 different data conditions specified by the nature of the relationship, form of the growth patterns, and the form of the effect modification from the exposure. Smallest average MSE for each data condition is bold.

Nature	Growth	Exposure	ME1	ME2	ME3	ME4	M1	M2	M3	M4
D	L	L	**16.3**	16.32	**16.3**	16.32	16.4	16.4	16.99	16.92
S	L	L	**23.13**	23.14	**23.13**	23.14	23.25	23.52	23.32	23.54
D	L	NL	**18.52**	18.53	**18.52**	18.53	18.57	18.57	18.76	18.7
S	L	NL	**22.57**	22.59	**22.57**	22.59	22.74	23.53	23.71	23.31
D	NL	L	38.98	**35.93**	38.98	**35.93**	38.99	35.94	39.2	36.07
S	NL	L	12.89	10.53	12.89	10.53	12.88	10.52	12.88	**10.51**
D	NL	NL	18.46	**13.02**	18.46	**13.02**	18.52	13.03	18.72	13.24
S	NL	NL	11.86	**10.45**	11.86	**10.45**	11.94	10.53	11.91	10.5

ME, Mixed Effect Model; M, Growth Mixture Model; D, Deterministic; S, Stochastic; L, Linear; NL, Non-Linear

Models 1, 3 assume linear mean; Models 2, 4 assume quadratic mean.

Models 1, 2 use random intercept; Models 3, 4 use random slopes.
